# Comprehensive Evaluation and Transcriptome Analysis Reveal the Salt Tolerance Mechanism in Semi-Wild Cotton (*Gossypium purpurascens*)

**DOI:** 10.3390/ijms241612853

**Published:** 2023-08-16

**Authors:** Zhen Peng, Abdul Rehman, Xiawen Li, Xuran Jiang, Chunyan Tian, Xiaoyang Wang, Hongge Li, Zhenzhen Wang, Shoupu He, Xiongming Du

**Affiliations:** 1 Zhengzhou Research Base, National Key Laboratory of Cotton Bio-Breeding and Integrated Utilization, School of Agricultural Sciences, Zhengzhou University, Zhengzhou 450001, China; pengzhen01@caas.cn (Z.P.); abdulpbg@gmail.com (A.R.); xiawensummer@163.com (X.L.); jiangxurann@163.com (X.J.); tcy130806@163.com (C.T.); wangxiaoyang198806@126.com (X.W.); lihongge@caas.cn (H.L.); 2 National Key Laboratory of Cotton Bio-Breeding and Integrated Utilization, Institute of Cotton Research, Chinese Academy of Agricultural Sciences, Anyang 455000, China; shenhuawzz@163.com; 3 National Nanfan Research Institute (Sanya), Chinese Academy of Agricultural Sciences, Sanya 572025, China

**Keywords:** *Gossypium purpurascens*, phenome, receptor-like kinase, salt tolerance evaluation, transcriptome, transcription factor, WGCNA

## Abstract

Elevated salinity significantly threatens cotton growth, particularly during the germination and seedling stages. The utilization of primitive species of *Gossypium hirsutum*, specifically *Gossypium purpurascens*, has the potential to facilitate the restoration of genetic diversity that has been depleted due to selective breeding in modern cultivars. This investigation evaluated 45 *G. purpurascens* varieties and a salt-tolerant cotton variety based on 34 morphological, physiological, and biochemical indicators and comprehensive salt tolerance index values. This study effectively identified a total of 19 salt-tolerant and two salt-resistant varieties. Furthermore, transcriptome sequencing of a salt-tolerant genotype (Nayanmian-2; NY2) and a salt-sensitive genotype (Sanshagaopao-2; GP2) revealed 2776, 6680, 4660, and 4174 differentially expressed genes (DEGs) under 0.5, 3, 12, and 24 h of salt stress. Gene ontology enrichment analysis indicated that the DEGs exhibited significant enrichment in biological processes like metabolic (GO:0008152) and cellular (GO:0009987) processes. MAPK signaling, plant-pathogen interaction, starch and sucrose metabolism, plant hormone signaling, photosynthesis, and fatty acid metabolism were identified as key KEGG pathways involved in salinity stress. Among the DEGs, including NAC, MYB, WRKY, ERF, bHLH, and bZIP, transcription factors, receptor-like kinases, and carbohydrate-active enzymes were crucial in salinity tolerance. Weighted gene co-expression network analysis (WGCNA) unveiled associations of salt-tolerant genotypes with flavonoid metabolism, carbon metabolism, and MAPK signaling pathways. Identifying nine hub genes (*MYB4*, *MYB105*, *MYB36*, *bZIP19*, *bZIP43*, *FRS2 SMARCAL1*, *BBX21*, *F-box*) across various intervals offered insights into the transcriptional regulation mechanism of salt tolerance in *G. purpurascens*. This study lays the groundwork for understanding the important pathways and gene networks in response to salt stress, thereby providing a foundation for enhancing salt tolerance in upland cotton.

## 1. Introduction

Climate change has profound effects on water resources, leading to the depletion of water supplies and the consequent salinization of soil [1]. Salinization is a major global abiotic challenge, affecting agricultural productivity and natural ecosystems [2]. Salt stress, characterized by elevated Na^+^ and Cl^−^ ion levels, adversely affects seed germination by increasing osmotic potential, reducing water absorption, and promoting ion uptake [3]. The worldwide concern is a reduction in cultivable land caused by rising sea levels, declining groundwater recharge, traditional irrigation methods, and increasing evapotranspiration. High soil salinity levels harm plant growth, development, and crop quality, making it crucial to develop salt-tolerant genotypes that maintain high yields under saline conditions [4].

Plants primarily respond and adapt to salt stress through various hormones and biosynthetic and signaling pathways controlled by gene expression regulation [5,6]. Transcription factors are crucial in regulating signal transduction and the transcription of numerous stress-related genes, including AP2/ERF, WRKY, MYB, NAC, and bZIP proteins [7,8,9,10]. Furthermore, carbohydrate metabolism and mitogen-activated protein kinase (MAPK) pathways also involve plant responses to salt stress [11]. The stimulation of receptor-like protein kinase (LRK) has been implicated in the salt stress response in conjunction with regulating the antioxidant system and the ABA-mediated signaling network [12]. Overexpression of RLK has been observed to enhance salt tolerance in rice by regulating stomatal patterns and ROS scavenging mechanisms [13]. The molecular pathways underpinning seed germination in response to salt stress are still unknown [14]. Therefore, it is important to identify metabolic pathways and genes that respond to salt stress to enhance the genetic resources of plants, enabling them to flourish in soils that are impacted by high salinity.

Advancements in sequencing technology and decreasing costs have propelled the field of plant science into the omics era. Various omics methodologies, such as phenomics, proteomics, transcriptomics, metabolomics, and genomics, have gained prominence in research [15,16]. Among these techniques, transcriptome sequencing, also known as RNA-Seq, has emerged as a powerful tool for studying gene expression patterns in both model and non-model plants, providing valuable insights into RNA-level gene expression [17]. Transcriptome analysis has played a pivotal role in identifying candidate genes and unravelling the biochemical, physiological, and molecular mechanisms underlying salt stress in various crops, including wheat [18,19], maize [20], rice [21], sorghum [22], and soybean [23]. Likewise, underlying molecular mechanisms of salt-tolerant genes *WRKY11*, *TaFBA1*, *BES1*, *R2R3-MYB*, and *GhLOG3* were detected in soybean, tobacco, Arabidopsis, and cotton [24,25,26,27,28]. Weighted gene co-expression network analysis (WGNCA) is a molecular biological network approach extensively employed to construct gene interaction relationships, identify regulatory genes, and forecast the role of unknown genes [29,30,31]. Earlier researchers identified hub genes and key regulatory pathways against salt tolerance with WGCNA and explored the mechanism of transcriptional regulation in Arabidopsis [32], wheat [33], maize [34], rice [35], and cotton [36]. 

Upland cotton (*Gossypium hirsutum* L.), a member of the Malvaceae family, is a globally significant crop accounting for 90% of textile fiber production. It is an allotetraploid species cultivated in over 80 countries, including semi-arid and tropical regions [37,38]. However, its moderate salt tolerance makes excessive salinity a major threat by reducing seed germination and limiting seedling growth, development, and productivity [39]. During the 17th century, *G. purpurascens* was identified as a perennial plant in various regions such as Brazil, Africa, China, Egypt, Congo, and India [40,41]. Initially, it was identified as a separate species [41,42], but it was later classified as a race of *G. hirsutum* [43]. However, subsequent studies did not place it among the geographical races of *G. hirsutum* [44]. There is a possibility that *G. purpurascens* has been naturally occurring in the South China Sea Islands for centuries without human intervention [45]. It is unknown where it came from, its evolutionary connection, when it was brought to these places, or how it was brought there. Structural variations played a significant role in the improvement and domestication of *G. purpurascens* [46]. Considering the significance of cotton, it is crucial to augment its salt tolerance by elucidating prospective genes through molecular investigations and genetic modifications. RNA-Seq differential expression analysis can serve as a valuable tool to discern salt tolerance genes, especially in wild progenitors, thereby facilitating the comprehension of the underlying mechanisms and pathways involved in salt tolerance [47]. In light of our previous research on the genome of *G. purpurascens* [48], it may contain salt-tolerant genes due to its evolution, genome diversification, domestication, and structural variations. Hence, high-throughput transcriptome sequencing was employed to investigate the gene expression profiles of primitive landraces of *G. purpurascens* subjected to salt stress and potential candidate genes were identified through WGCNA analysis. The findings of this study have the potential to provide valuable insights into the molecular mechanisms underlying salt stress responses in various cotton species and contribute to our understanding of salinity stress in other crop plants.

## 2. Results

### 2.1. Comprehensive Evaluation of Salt Tolerance of 45 G. purpurascens at the Seedling Stage

A total of 45 varieties of *Gossypium purpurascens*, along with one control variety of *Gossypium hirsutum*, were evaluated for salinity tolerance at the seedling stage. The seedlings were subjected to two different NaCl concentrations (0.0% and 0.4%) and data were recorded at various time points (0 h, 0.5 h, 3 h, 12 h, and 24 h) after the onset of salt stress. Several parameters related to plant growth and ion concentrations were assessed, including fresh root weight, dry root weight, fresh shoot weight, dry shoot weight, root-shoot ratio, relative water content, and ion-selective coefficients for Na, K, and Mg ions. Statistical analyses were performed on the collected data, including standard deviation, coefficient of variation, principal component analysis, and correlation analysis (Supplementary Table S1).

**Figure 1:**
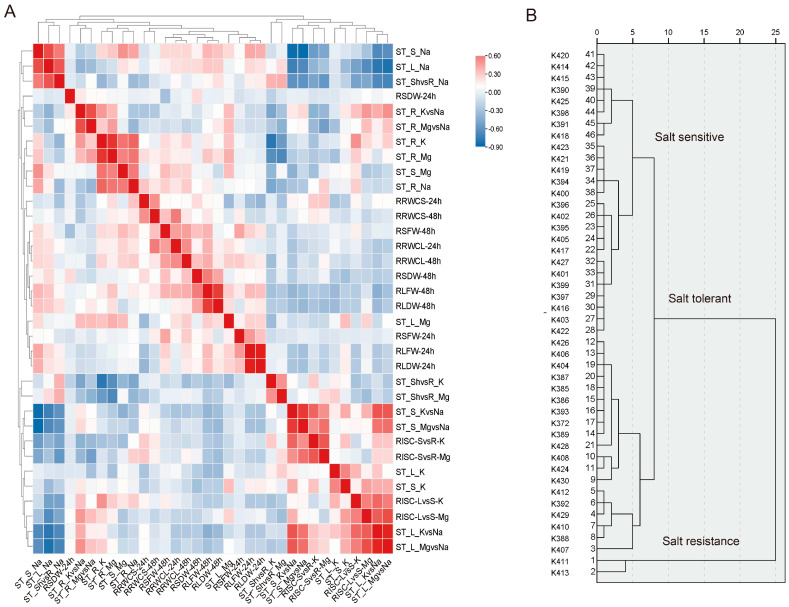
Forty-five genotypes of *G. purpurascens* for the salt tolerance identification. (**A**) Correlation of 34 physiological and biochemical traits between biomass and ionome indexes. (**B**) Based on the comprehensive salt tolerance index (CSTI), clustering of 45 genotypes of *G. purpurascens* and one *G. hirsutum* (K372, salt tolerance control, germplasm Zhong9807). Three major groups were identified: Salt-resistant germplasm, salt-tolerant germplasm, and salt-sensitive germplasm.

The correlation analysis revealed positive and negative associations between traits (Figure 1A). Notably, Na concentration in salt-treated shoots showed a strong positive correlation with Na concentration in salt-treated leaves and the shoot-to-root ratio. Similarly, positive correlations were observed between roots’ and stems’ Na, K, and Mg concentrations. In contrast, significant negative correlations were observed between the dry weight and root-shoot ratio after 24 h (RSDW-24 h) and between most of the traits. The biomass and relative water content reduction of cotton seedlings were more pronounced in salt-sensitive genotypes than in salt-resistant genotypes. Additionally, there were significant correlations observed between salt tolerance-related traits.

Moreover, variables were arranged according to Euclidean distance hierarchical cluster analysis (Figure 1B). The variables were categorized according to their patterns of correlation. The classification of the 46 cotton genotypes into three groups, namely salt-sensitive, salt-tolerant, and salt-resistant, was determined using the comprehensive salt tolerance index (CSTI) (Figure 1B, Supplementary Table S1). The genotypes K411 and K413 demonstrated the highest values for CSTI, indicating their salt resistance, whereas 25 genotypes exhibited the lowest values, indicating their salt sensitivity. The remaining nineteen genotypes were classified as exhibiting salt tolerance. The control genotype *G. hirsutum* K732 was ranked in the salt-tolerant group.

### 2.2. The Phenome of Two Genotypes with Contrasting Differences in Salt Tolerance Showed Significant Differences under Salinity Stress

**Figure 2:**
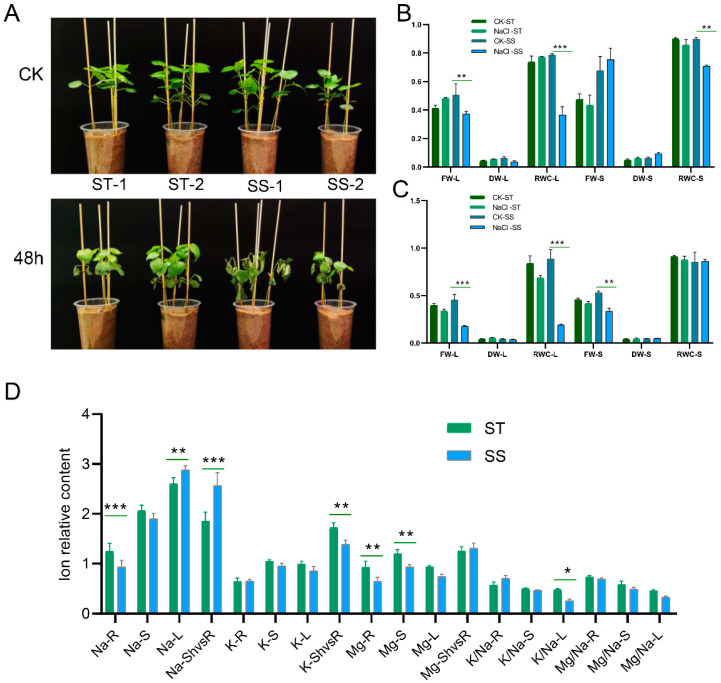
Differences in salt tolerance between two varieties of *G. purpurascens* after NaCl treatment: NY2 (salt-tolerant; ST) and GP2 (salt-sensitive; SS) physiological and biochemical characteristics. (**A**) Response of salt-tolerant and salt-sensitive genotypes compared to control 48 h after 0.4% NaCl treatment. (**B**) Comparison of fresh weight, dry weight, and relative water content of leaves and stems of salt-tolerant and salt-sensitive materials after 24 h of 0.4% NaCl treatment. (**C**) Comparison of fresh weight, dry weight, and relative water content of leaves and stems of salt-tolerant and salt-sensitive materials after 48 h of 0.4% NaCl treatment. (**D**) Comparison of Na, K, and Mg contents in roots, stems, and leaves and their ratios in salt-tolerant and salt-sensitive genotypes after 48 h. There were three biological replicates of the data, with significant differences determined by two-tailed *t*-test; * *p* < 0.05; ** *p* < 0.01; *** *p*< 0.001.

Further study focused on one salt-tolerant (ST) variety, K386 (NY2), and one salt-sensitive (SS) genotype, K396 (GP2), which were screened and selected based on biomass and ionome indexes. These varieties were subjected to a salt treatment of 0.4% NaCl for different durations (0.5 h, 3 h, 12 h, 24 h, and 48 h) under the same growth conditions (Figures S1 and S2). The SS genotype exhibited wilting symptoms after 48 h of salt treatment, while the ST genotype showed better performance in terms of fresh weight (FW), dry weight (DW), and relative water content (RWC) of the leaves after 24 h of salt treatment (Figure 2A). The FW, DW, and RWC of the stems were higher in the SS genotype compared to the ST genotype (Figure 2B). A significant reduction in FW, DW, and RWC of the leaves was observed in the salt-treated salt-sensitive genotype compared to the salt-tolerant genotype after 48 h of NaCl treatment (Figure 2C). The salt-tolerant genotype had higher Na content in the roots and stems (Figure 2D), while the salt-sensitive genotype exhibited higher Na concentration in the leaves and a higher shoot-to-root ratio. The ST genotype also showed higher K and Mg content in the roots, stems, leaves, and shoot-to-root ratio. Additionally, higher K/Na and Mg/Na ratios were observed in various parts of the ST genotype, indicating its greater salt tolerance compared to that of the SS genotype (K386/NY2 vs. K396/GP2).

### 2.3. RNA-Seq Analysis

**Figure 3:**
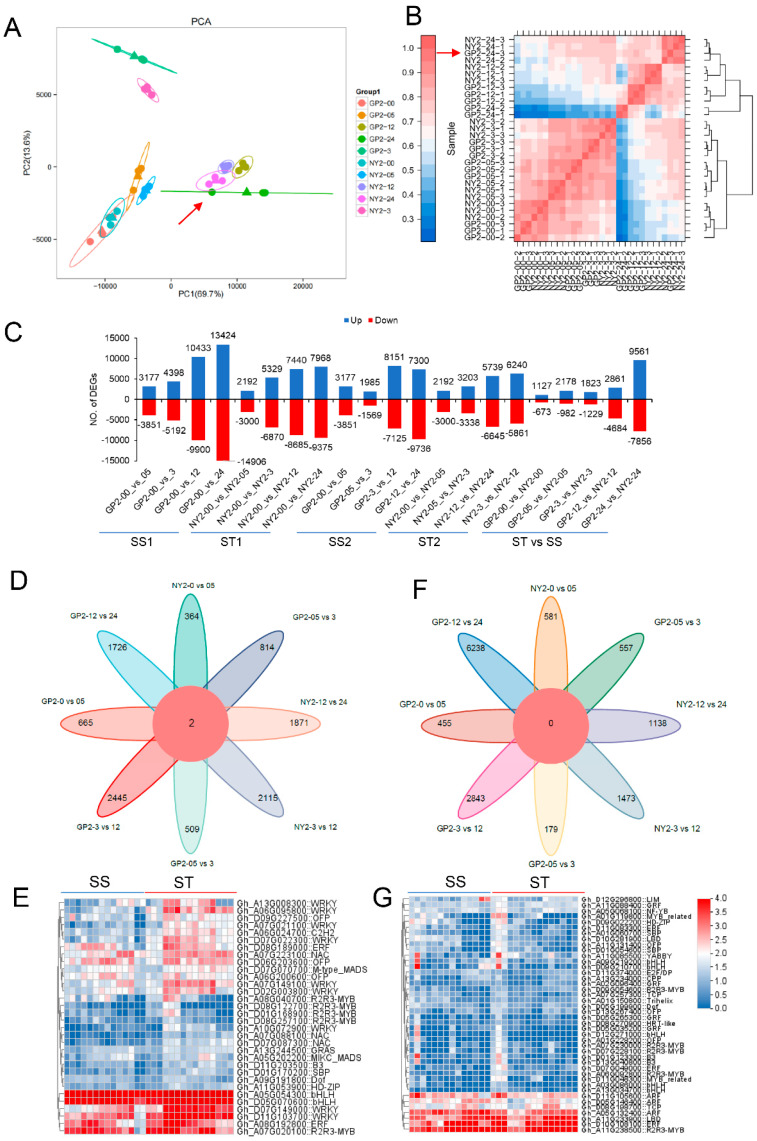
Overview of the transcriptome analysis of 30 samples of *G. purpurascens*. (**A**) Principal component analysis of the genotypes with treatment (red arrow means GP2-24-3 slightly outlier). (**B**) A heatmap depicting the results of sample correlation analysis based on transcriptome expression after NaCl treatment (it was found that the sample GP2-24-3 was not good (red arrow), and the differential expression gene (DEG) analysis was removed). (**C**) Number of differentially expressed genes in two cotton genotypes and all of their comparison groups after salt treatment. (**D**) Common and specific upregulated differentially expressed genes (DEGs) in eight different comparison groups. (**E**) Common and specific downregulated differentially expressed genes (DEGs) in eight different comparison groups. (**F**) FC heat map of upregulated TFs in NY2-0 vs. 05 DEG. (**G**) FC heatmap of downregulated TFs in NY2-0 vs. 05 DEG.

Total RNA was collected from leaf tissues of cotton seedlings subjected to different salinity stress durations (0, 0.5, 3, 12, and 24 h). The RNA samples were then subjected to transcriptome sequencing to investigate the molecular mechanisms underlying salt tolerance in cotton seedlings. A total of 29 cDNA libraries were generated, and high-throughput sequencing generated 221.06 Gb of raw sequencing data, with a range of 39,604,862 to 58,894,078 raw reads per library. After quality filtering, the number of clean reads per library ranged from 19,802,431 to 29,447,039, with 50% having clean reads. The quality of the sequencing data was assessed based on Q20 and Q30 values, which ranged from 99.18% to 94.89%, indicating high-quality sequencing (Supplementary Table S2). The range of mapping ratios for the clean reads to the cotton reference genome was between 94.88% and 98.84%. A successful mapping rate of 98.74% to 98.81% was achieved among the clean reads. Following quality control, over 1.98 million clean reads were obtained for each sample, with GC contents ranging from 43.27% to 44.54%. The percentage of uniquely mapped reads ranged from 90.16% to 93.89%, while the percentage of multi-mapped reads ranged from 4.71% to 7.63%. The majority of the aligned sequences were found to be located in exon regions (85.42%), while a smaller proportion were mapped to intron regions (4.59%) and intergenic regions (9.99%). The mapping results were further analyzed for alternative splicing prediction, gene structure optimization, and novel gene discovery. A comprehensive set of 10,305 genes were identified, with 6701 annotated with putative functions. The gene expression data underwent principal component analysis (PCA), identifying eight primary groups and one outlier (Figure 3). The first and second eigenvectors explained 67.7% and 13.6% of the total variation, respectively. The PCA analysis showed that 83.3% of the variations could be attributed to the different treatments. The genotypes GP2 and NY2, when subjected to salt treatment, exhibited distinct separation from the control and treatment groups. This observation suggests significant gene expression variations between the salt-treated and control plants. Outliers were identified in the GP2-24 group (Figure 3A). The three biological replicates for each time point and genotype showed close clustering, indicating acceptable variance within the replicates. The analysis of correlation and dendrogram clustering identified six distinct clusters of samples, characterized by significant correlations within each cluster, i.e., GP2-00-2, GP2-00-1, GP2-00-3, NY2-00-2, NY2-00-1, and NY2-00-3 were clustered in the first group whereas NY2-05-3, NY2-05-1, NY2-05-2, GP2-05-1, GP2-05-2, and GP2-05-3 were assigned to the second group (Figure 3B), while GP2-3-2, GP2-3-1, GP2-3-3, NY2-3-3, NY2-3-1, and NY2-3-1 were in the third cluster. Interestingly, all three of these groups showed strong correlations with each other. These findings suggested that the gene expression patterns of the two cultivars remained consistent under normal conditions and at timepoints 0.5 and 3 h after salt treatment.

Clear contrasts in gene expression were observed between SS and ST genotypes at 12 and 24 h, indicating significant transcriptomic shifts as GP2-24-1 and GP2-24-2 were enlisted in the fourth cluster while GP2-24-1, NY2-24-2, NY2-24-1, and NY2-24-3 were observed in the sixth cluster. GP2-12-2, GP2-12-1, GP2-12-3, NY2-12-3, NY2-12-1, and NY2-12-2 were assigned to the fifth cluster. Due to the significant distances between GP2-24-1 and GP2-24-2 and GP2-24-3, the sample was removed from subsequent differential expression analysis. The analysis indicated that salinity stress affects seedling growth differently in salt-tolerant and salt-sensitive cultivars, highlighting the significant differences between the two accessions. Pearson’s correlation analysis further indicated that the salt-tolerant cultivar may provide valuable insights into the mechanisms underlying salt tolerance. Differential expression analysis and identification of new genes were also performed using the filtered high-quality reads.

### 2.4. Identification of DEGs Using RNA-Seq

In this study, differentially expressed genes (DEGs) were identified based on a significant value (*p*-value < 0.05) representing a greater than twofold change in transcriptional expression compared to the control (0 h time point) under salt treatment. The number of DEGs at different time intervals (0.5, 3, 12, and 24 h) in both GP2 and NY2 genotypes were compared to the control to understand the global transcriptional changes in response to salt treatment. In the GP2 genotype, the number of DEGs increased with increasing salt stress. At 0.5, 3, 12, and 24 h, 7028, 9590, 20,333, and 28,330 DEGs were detected, respectively. Similarly, in the NY2 genotype, 5192, 12199, 16125, and 17,343 DEGs were identified at 0.5, 3, 12, and 24 h, respectively (Figure 3C). Pairwise comparisons at 3/12 and 12/24 h revealed that GP2 showed a higher number of DEGs (15,276 and 17,036, respectively) compared to NY2 (12,101 and 12,384, respectively), indicating more advanced regulation in GP2 under salt stress. Conversely, NY2 showed higher regulation with 6541 DEGs in the 0.5/3 h comparison group compared to GP2.

Furthermore, DEGs were simultaneously analyzed between the two genotypes to identify salt tolerance-related genes. A total of 1127, 2178, 1823, 2861, and 9561 DEGs were identified as upregulated in GP2 compared to NY2 at 0, 0.5, 3, 12, and 24 h, respectively. In contrast, a total of 673, 982, 1229, 4684, and 7856 DEGs were observed to be downregulated in GP2 when compared to NY2 during the respective time intervals. The comparison between GP2-24 and NY2-24 exhibited the highest number of DEGs, indicating a particular gene expression pattern between the two genotypes at 24 h. A reverse trend was observed in the GP2-12 vs. NY2-12 comparison group. Analysis of upregulated genes at various time intervals in both genotypes revealed two common genes in eight comparison groups (Figure 3D), suggesting the involvement of these genes in response to salinity tolerance mechanisms. But no common downregulated differentially expressed gene was detected in any of the comparison groups (Figure 3F).

A heat map was generated to visualize the upregulated transcription factors (TFs) in both salt-sensitive (SS) genotype GP2 and salt-tolerant (ST) genotype NY2 (Figure 3E). The heat map revealed that bHLH (Gh_A05G054300, Gh_D05G070600), WRKY (Gh_D11G103700), ERF (Gh_A08G192800), and R2R3-MYB (Gh_A07G020100) are principal upregulated TFs that play a vital role in salinity tolerance. Conversely, R2R3-MYB (Gh_A11G238500), ERF (Gh_D10G108100), LBD (Gh_A11G233900), and ARF (Gh_D11G105600) are downregulated TFs associated with salt tolerance in cotton (Figure 3G, Supplementary Table S3). Overall, the analysis of DEGs provided insights into the global transcriptional changes, identified salt tolerance-related genes, and highlighted key transcription factors involved in response to salinity stress in cotton.

### 2.5. Identification of DE Salt Tolerance-Related Genes and Key KEGG Pathways

**Figure 4:**
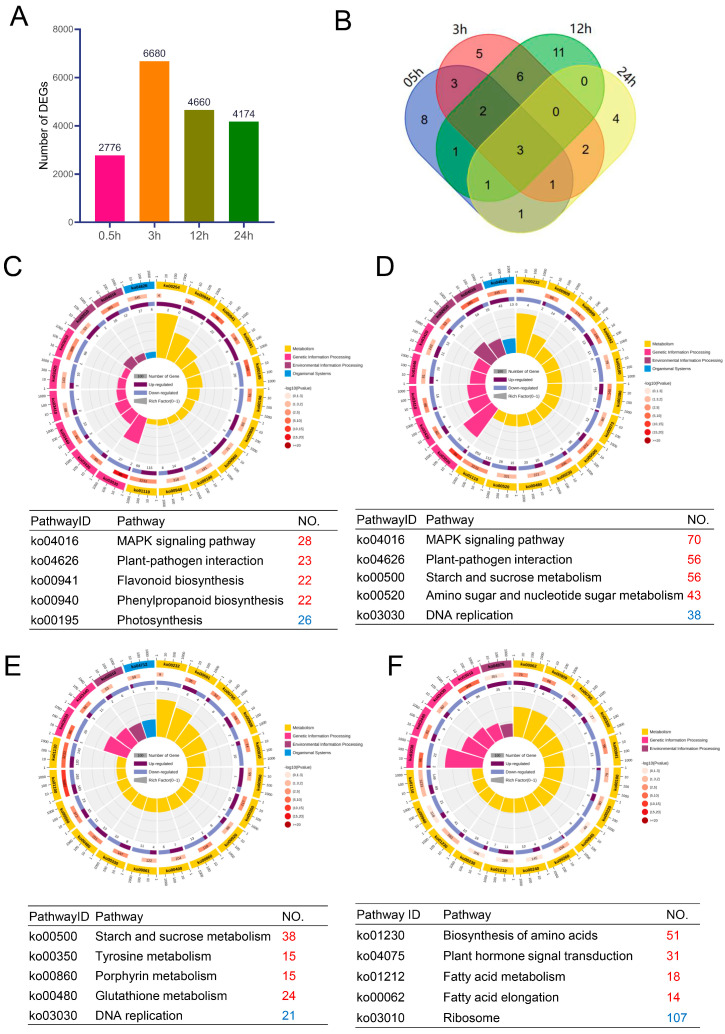
Number of differentially expressed genes associated with salt tolerance and enrichment analysis of metabolic pathways. (**A**) Comparison of the number of DEG in salt tolerance-related differentially expressed genes at four time points. (**B**) Venn diagram illustrating the number of overlapping DEGs between four time intervals. Overlapping regions indicate co-expressed DEGs among different datasets, and numbers in only one circle represent DEGs expressed in only one library. (**C**) Top 5 KEGG enrichment pathways for salt tolerance-related differentially expressed genes at 0.5 h; (**D**) 3 h; (**E**) 12 h; (**F**) 24 h. Red represents the number of upregulated genes, while blue represents the number of downregulated genes.

Under established conditions, we identified genotype-specific and salt-responsive differentially expressed genes (DEGs) related to salt tolerance in two cotton genotypes. A total of 2776, 6680, 4660, and 4174 salt-tolerant DEGs were identified at 0.5, 3, 12, and 24 h, respectively (Figure 4A, Supplementary Table S3). Three common DEGs associated with salt tolerance were identified across all four time intervals (Figure 4B). A four-way Venn diagram suggests that the DEGs were genotype-specific and salt-responsive. The highest number of salt-tolerant genes was observed after 12 h of exposure to 0.4% NaCl. Comparative analysis between the two genotypes revealed specific DEGs, with NY2 showing more common DEGs across different time intervals than GP2. NY2-00 exhibited a consistent pattern of DEGs at 3, 12, and 24 h, while GP2 showed genotype-specific DEGs at each time point. These findings provide insights for selecting screening criteria for salt tolerance genes.

### 2.6. Analysis of DEGs Based on the Gene Ontology (GO) and the Kyoto Encyclopedia of Genes and Genomes (KEGG)

This study employed GO enrichment analysis to categorize the functional roles of the DEGs observed in seedlings under salinity stress conditions in both genotypes (Supplementary Table S3). A total of 3615 DEGs were identified, associated with diverse biological processes, molecular functions, and cellular components. After 24 h of salt treatment, gene ontology functional types showed the highest representation among biological processes. The analysis indicated significant enrichment in NY2 samples compared to GP2 samples across different time points.

Furthermore, a KEGG pathway enrichment analysis was conducted to explore the roles of DEGs concerning salinity tolerance. A total of 2804 salt-tolerant DEGs were found to be involved in KEGG enrichment pathways. At different time intervals, various pathways showed enrichment, including MAPK signaling, plant-pathogen interaction, starch and sucrose metabolism, and biosynthesis of amino acids (Figure 4C–F). The metabolic pathways were consistently prominent in all periods. Heat map analysis of the upregulated MAPK signaling pathway revealed specific (*PP2C* (Gh_D03G014500), ERF1 (Gh_A02G045500) and *ACS1* (Gh_D11G099900)) and downregulated genes (*SPCH* (Gh_D12G271000) and *CHB* (Gh_A03G238200)) associated with salt tolerance (Figure S4). *KCS1* (Gh_D06G142800) was upregulated in all comparison groups except the GP2-00 vs. GP2-05 group in plant-pathogen interaction. *AP1* (Gh_D05G091100), *ABI1* (Gh_A13G222500), and *ABI2* (Gh_D13G222500) were upregulated and *PIF3* (Gh_D11G310300), *AHK4* (Gh_D12G171900, Gh_A12G175000), and *XTH22* (Gh_D05G318000) were downregulated in plant hormone signal transduction. Similarly, *KCS1* (Gh_D08G142800) was upregulated in all the comparison groups except the GP2-00 vs. GP2-05 group. *TPS* (Gh_D08G093700) was upregulated in starch and sucrose metabolism, while *INV* (Gh_A04G061600) was downregulated. Overall, the analysis indicated that MAPK signaling, plant-pathogen interaction, starch and sucrose metabolism, and biosynthesis of amino acids pathways played crucial roles in regulating salt tolerance at the seedling stage of *G. purpurascens*.

### 2.7. TF, RLK, CAZy, and Other Key Genes Involved in Salinity Tolerance

**Figure 5:**
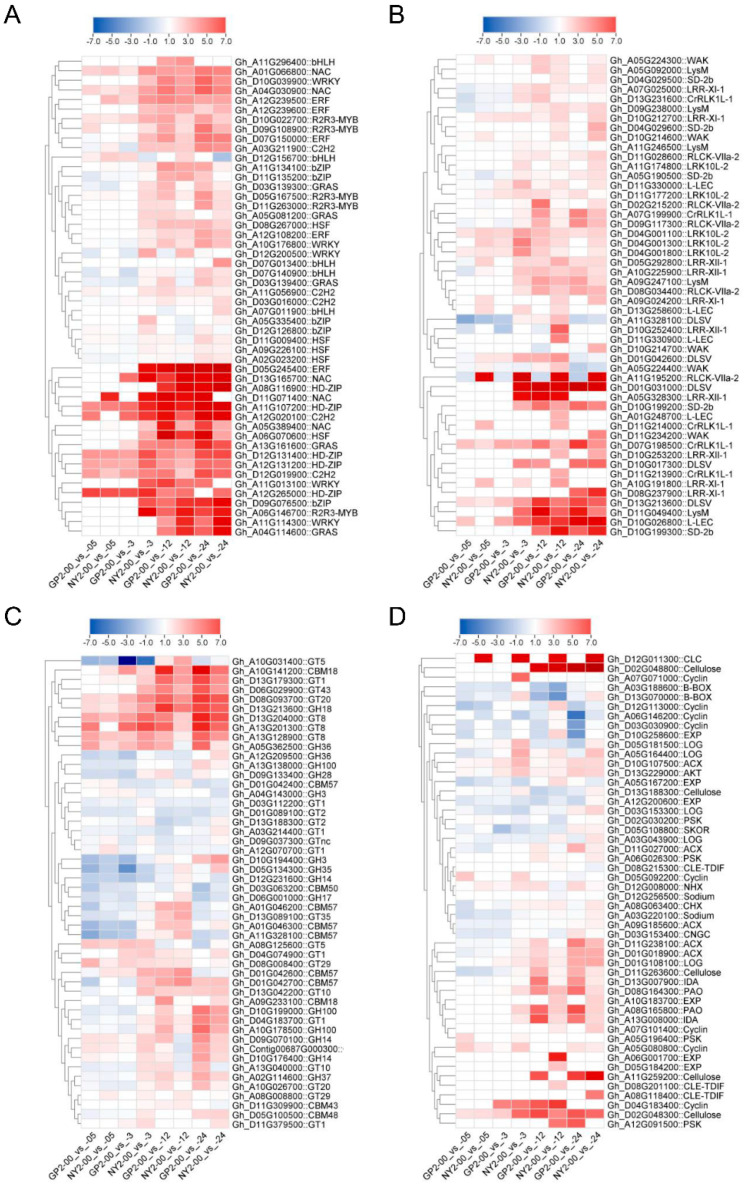
Comparison of the heat map of differently expressed genes related to salinity tolerance gene families and transcription factors in transcriptomic data. (**A**) TF, (**B**) RLK, (**C**) CAZy, (**D**) others.

A total of 924 genes belonging to 25 transcription factor (TF) families were identified in the DE salt tolerance-related genes. These TF genes showed diverse expression patterns, indicating their various roles in response to salinity (Figure 5A, Supplementary Table S4). WRKY, R2R3-MYB, bHLH, NAC, bZIP, and HD-ZIP showed 75, 70, 69, 62, 56, and 50 DEGs, respectively. Specifically, 25 types of receptors, like kinase genes, were identified in 501 DEGs (Supplementary Figure S5B). The heat map highlighted the upregulation of TFs such as HD-ZIP (*ATHB-7*) (Gh_A11G107200), NAC (*NAC056*) (Gh_D13G165700), ERF (*ERF114*) (Gh_D05G245400), R2R3-MYB (*MYB102*) (Gh_A06G146700) and C2H2 (*ZAT10*) (Gh_A12G020100), which were associated with salinity tolerance. Additionally, bZIP (*bZIP1*) (Gh_D09G076500) and HSF (*HSFC1*) (Gh_A06G070600) showed upregulation at multiple time intervals, while *GRAS* (Gh_A13G161600) demonstrated upregulation at 12 and 24 h.

The expression analysis of the receptor-like kinase family (RLK) revealed that GP2 and NY2 exhibited tolerance against salinity at 3, 12, and 24 h (Figure 5B, Supplementary Table S4). Upregulation of genes belonging to the *SD-2b* (Gh_D10G199300, Gh_D10G199200), *L-LEC* (Gh_D10G026800), *DLSV* (Gh_D01G031000), *LysM* (Gh_D11G049400), and *CrRLK1L-1* (Gh_D07G198500) subfamilies was observed. Notably, *RLCK* (Gh_A11G195200) was identified as a novel gene, upregulated in the NY2 genotype but downregulated in the GP2 genotype. Carbohydrate-active enzymes (CAZymes), which are involved in carbohydrate metabolism, also showed distinct expression patterns. Genes such as *GH36* (Gh_A05G362500), *GT8* (Gh_D13G204000), *GH18* (Gh_D13G213600), *GT20* (Gh_D08G093700), and *CBM18* (Gh_A10G141200) were consistently upregulated across different comparison groups (Figure 5C, Supplementary Table S4). *GT1* (Gh_D13G179300) and *GT43* (Gh_D06G029900) were upregulated at 3, 12, and 24 h. Other gene families, such as cellulose synthase-like protein *G2* (Gh_D02G048800) and *EXP* (Gh_A12G200600), exhibited specific expression patterns concerning salinity tolerance groups (Figure 5D, Supplementary Table S4). Among the TFs, ERF had the highest number of DEGs (105) and was identified as a major contributing factor to salinity tolerance in both genotypes (Figure S5A). WRKY, R2R3-MYB, bHLH, NAC, bZIP, and HD-ZIP were prominent TF families related to salt tolerance. Receptor-like kinase genes, including *DLSV*, *LRR-XI-1*, *CrRLK1L-1*, *LRR-III*, *LRK10L-2*, and *L-LEC*, were crucial in salinity tolerance. The aforementioned results underscore the notable regulatory functions of identified transcription factors in the cotton plant’s response to salt stress. The salt-tolerant cultivar NY2 responded faster to salt stress than the salt-sensitive cultivar GP2.

### 2.8. Gene Co-Expression Network Analysis Identifies Specific Modules Related to Salt Stress in G. purpurascens

Weighted gene co-expression network analysis (WGCNA) explored the gene regulatory network associated with salt stress response in GP2 and NY2 genotypes. Initially, 216,006 genes exhibiting differential expression between adjacent developmental stages of GP2 and NY2 were identified through transcriptome analysis. These genes were filtered based on their fold change (|log2FC| > 1) and false discovery rate (FDR < 0.05). Genes with FPKM values less than five were excluded from the analysis. The WGCNA package was used to compute weight values for network construction, and a “soft threshold” of 12 was established to ensure a scale-free network distribution.

**Figure 6:**
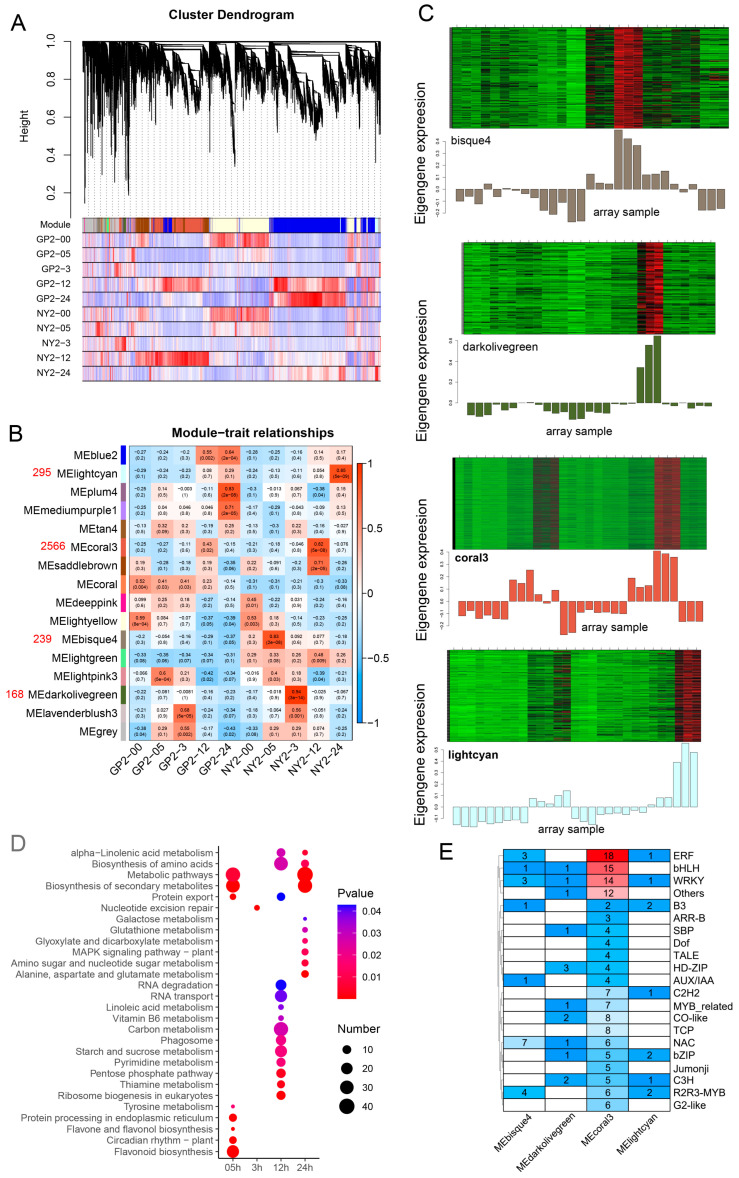
Weighted gene co-expression network analysis (WGCNA) construction and core gene identification. (**A**) Gene clustering trees and module division; (**B**) Module-trait correlation heat map (The red number represents the number of genes in that module); (**C**) The expression levels of all genes and corresponding MEs in different modules in each sample; (**D**) Gene KEGG enrichment pathway for four distinctive modules. (**E**) Statistics on the number of TFs in genes of the four significance modules.

A gene clustering tree was constructed by utilizing the correlation between distinct gene expression levels. The dynamic cutting tree method was subsequently employed to partition the branches and produce distinct modules (Figure 6A). Sixteen gene co-expression modules were obtained with shared expression patterns (75% similarity) (Figure 6B, Supplementary Table S5). Among these modules, ME coral3 had the highest number of genes (2566), followed by ME lightcyan (295), ME bisque4 (239), and ME darkolivegreen (168). Grey modules represented unassignable genes.

Correlation analysis was performed to identify modules significantly associated (*p* < 0.01) with salt stress treatments. The ME coral3 module demonstrated the highest number of genes (2566), followed by MElightcyan (295), MEbisque4 (239), and MEdarkolivegreen (168) modules (Figure 6B). The modules ME lightcyan, ME coral3, ME bisque4, and ME darkolivegreen showed significant correlations and were selected for further analysis. Some modules showed high specificity with samples, such as ME darkolivegreen with NY2-3 and ME lightgreen with NY2-24, demonstrating strong positive correlations. A graph displaying the log10 FPKM values of genes within each module and eigengene expression values was generated to understand each co-expressed module’s uniqueness and expression patterns across datasets (Figure 6C). The gene expression levels in all four identified modules were elevated under salt-treated NY2, confirming the previously observed positive correlations.

KEGG pathway analysis revealed that metabolic and biosynthesis of secondary metabolites were the most significantly enriched pathways, followed by flavonoid biosynthesis at 0.5 h of salt treatment (Figure 6D). At 12 h, biosynthesis of amino acids and carbon metabolism were predominant pathways. The presence of differentially expressed genes (DEGs) related to metabolic pathways and biosynthesis of secondary metabolites further emphasized their role in salinity tolerance. Moreover, the four selected modules contained 7, 10, 22, and 7 transcription factors, respectively (Figure 6E). DEGs for the WRKY transcription factor were observed in each selected module. The ME coral3 module, with the highest number of ERF, bHLH, and WRKY transcription factors, confirmed its key role in salinity tolerance. WGCNA identified gene co-expression modules associated with salt stress response in GP2 and NY2 genotypes. These modules exhibited specific expression patterns and were enriched in relevant pathways and transcription factors, shedding light on the regulatory mechanisms underlying salt tolerance.

### 2.9. Identification of Highly Connected and Central Hub Genes

To identify pivotal hub genes, a co-expressed module network was constructed. The size of the node circle in the network indicates the number of genes that interact with a specific node, reflecting the degree of connectivity. ME (module eigengene) expression levels were used to examine gene expression within co-expression modules, representing the overall expression levels of each module. The weights for each gene were computed by WGCNA using measures of gene importance, such as lower *p*-values and modular membership. Genes with stronger treatment associations were assigned higher weight values. Four selected modules yielded 14 hub genes based on their degree of connectivity (Supplementary Table S5).

**Figure 7:**
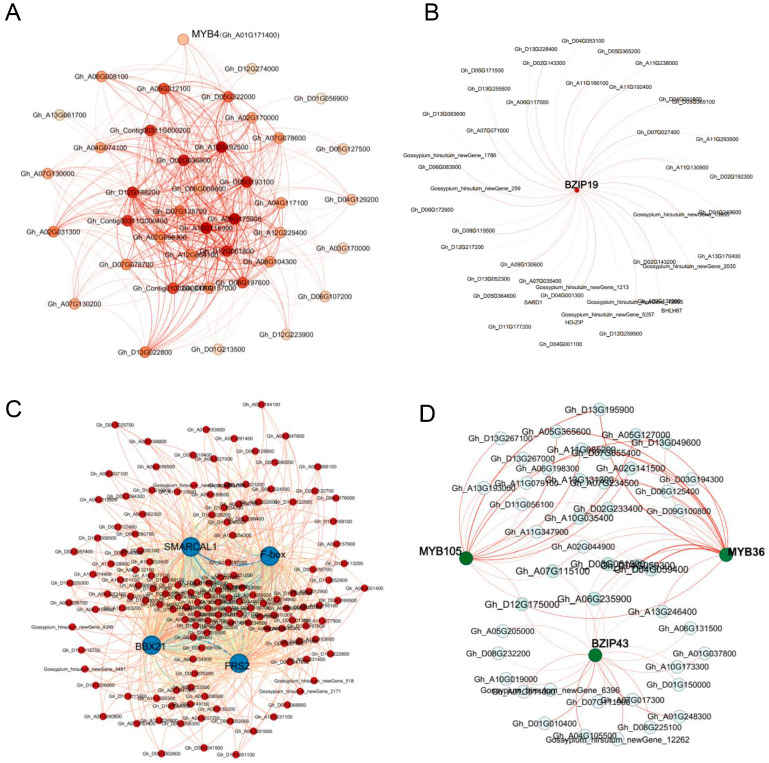
Gene co-expression network within specific modules associated with samples (tissues) after stress with salt-tolerant materials. Co-expression network within the ME bisque3 module (**A**); the ME darkolvegreen module (**B**); the ME coral3 module (**C**); the ME lightcyan module (**D**). Detailed co-expression information is provided in Supplementary Table S5.

Within the ME bisque3 module, MYB4 (Gh_A01G171400) was identified as the main differentially expressed gene (DEG) in the co-expression network. This module had 23 associated genes (Figure 7A, Supplementary Table S5A). In the ME darkolivegreen module, bZIP19 (Gh_A07G196600) was detected as the key regulator among 42 selected genes (Figure 7B, Table S5B). The ME coral13 module’s co-expression network included FRS2 (Gh_D07G203100), SMARCAL1 (Gh_D06G130300), BBX21 (Gh_D05G189200), and F-box (Gh_A04G064800) from 393 selected genes (Figure 7C, Table S5C). Finally, the ME lightcyan module had MYB105 (Gh_A13G181900), MYB36 (Gh_D09G156400), and bZIP43 (Gh_A07G196600) as key genes among the 40 identified genes (Figure 7D, Table S5D).

### 2.10. qRT-PCR Validation of RNA-Seq Data

**Figure 8:**
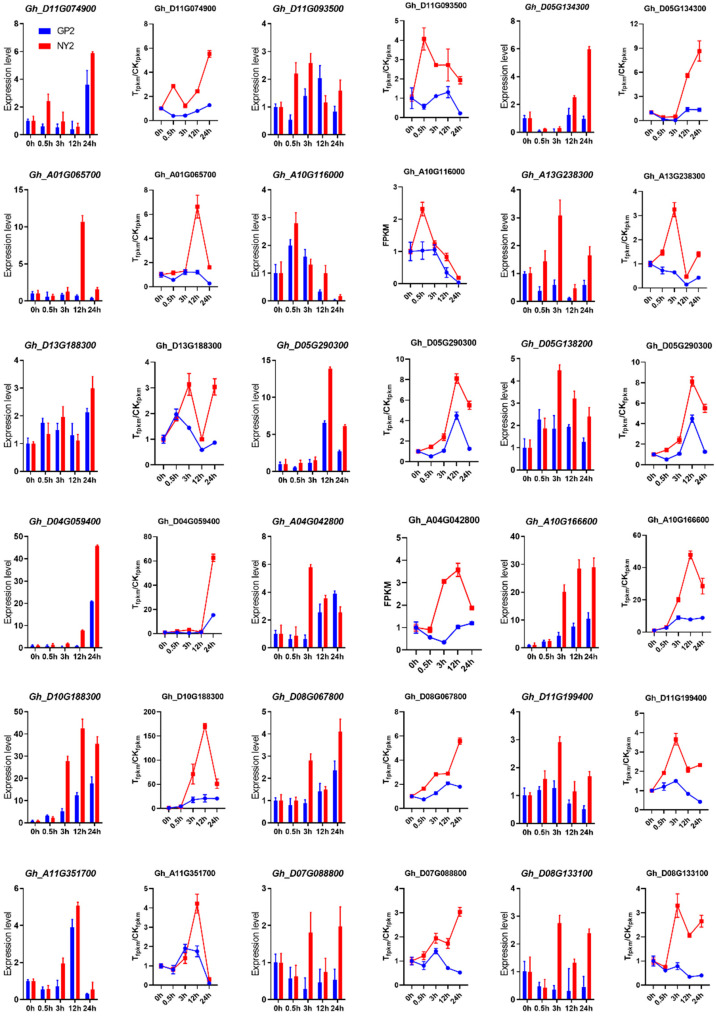
Quantitative real-time PCR validation of RNA-Seq data. The bar chart depicts qRT-PCR results, while the line graph represents RNA sequencing data.

Real-time PCR analysis was performed on eighteen randomly selected genes identified as significantly differentially expressed genes (DEGs) to validate the gene expression profiles obtained from the transcriptome data. This analysis aimed to compare the results with the corresponding transcriptome data, as shown in Figure 8. The primer sequences for these genes can be found in Supplementary Table S6. The expression levels of the chosen genes displayed notable alterations at each time interval after salinity treatment in the NY2 cultivar in contrast to the GP2 cultivar, suggesting their susceptibility to salt. Some genes showed higher expression levels at specific intervals, while others displayed upregulation and downregulation at multiple intervals, such as Gh_D05G134300 and Gh_D05G138200. Gh_A10G116000 showed downregulation at 0.5, 3, 12, and 24 h in both qRT-PCR and RNA-seq data. We also identified upregulation of the Gh_A10G166600 gene and its homologous Gh_D10G188300 in *G. purpurascens* under salt stress conditions when exposed to 0.4% NaCl. These genes belonged to the cellular component “mitochondrion” according to GO annotation (GO:0005739), and PFAM annotation revealed their association with LEA5 (Late embryogenesis abundant protein).

The results of this study demonstrated a significant positive correlation between the gene expression levels obtained through qRT-PCR and RNA-seq approaches. This finding confirms the reliability of the RNA-seq data used in the research. Furthermore, the hub genes identified by WGCNA exhibited differential expression under salt stress conditions compared to the expression levels in control seedlings. This observation implies that the identified hub genes may have a significant impact on the cellular response to salt stress.

## 3. Discussion

Plants have developed an advanced mechanism for altering their physiological and metabolic states in response to environmental variations. Salinity stress significantly challenges crop productivity, affecting various important agricultural crops such as maize, barley, wheat, rice, and cotton [49,50,51,52,53]. Cotton is a leading fiber crop around the globe with vital economic importance in the textile industry, particularly vulnerable to the adverse impacts of soil salinity. While cotton is a pioneer crop with some moderate salt tolerance, it is insufficient in addressing the growing problem of salinization. Therefore, to address the issue of cotton cultivation in alkaline soil, it is imperative to expand the selection range of germplasm resources, identify wild and semi-wild cotton lines with excellent salt tolerance traits, uncover genes and markers related to salt tolerance, and utilize molecular marker-assisted breeding or genetic engineering to develop salt−tolerant varieties. This is a critical approach to mitigating the challenge of growing cotton in saline soil.

### 3.1. Germplasm of G. purpurascens Collected from South China Sea Islands in China Exhibited Strong Salt Tolerance

In our study, we assessed the impact of salt stress on different cotton genotypes collected from the South China Sea Islands by evaluating various phenotypic and physio-biochemical attributes, including seedling growth parameters, relative water content, and the distribution of Na^+^ and K^+^ ions in shoot and root tissues. The results indicated that the GP2 genotype was significantly more affected by salt stress than the NY2 genotype. These findings validate the divergent interactions exhibited by specific cotton genotypes when subjected to saline conditions, as reported in previous studies [54,55]. Ionome profile analysis further revealed that the sensitive genotypes illustrated higher ratios of Na^+^ to K^+^ in their systems compared to the tolerant genotypes. This ion distribution pattern in plant tissues is significant because it is crucial in salt tolerance mechanisms [56]. The concentrations of Na^+^ and K^+^ ions and their respective ratios are important parameters in understanding plant responses to salt stress. Under salt stress conditions, the NY2 genotype exhibited a preferential accumulation of K+ and reduced accumulation of Na^+^, thereby maintaining shoot Na^+^/K^+^ ratios. Due to the distinct nature of the wild species, it is reasonable to consider that a Na+ extrusion from the root cells, coupled with an increased salt exclusion from the shoots, could potentially support the hypothesis in favor of wild cotton. The salt tolerance exhibited by wild cotton can be understood within the framework of anticipated evolution. This characteristic potentially reinforces the proposed evolutionary strategy found in wild plants adapted to saline environments. These observations aligned with prior rice and chickpea research [57,58,59]. According to Flowers, et al. [60], salt-tolerant genotypes maintain ion homeostasis by utilizing specific mechanisms such as ionic pumps, antiporters, and carrier proteins on cellular membranes. These mechanisms enable the exclusion and compartmentation of toxic ions, particularly shoot Na+, thus preventing toxicity in high salt concentrations [60,61]. By analyzing the ion distribution and ratios, our study provides valuable insights into the mechanisms underlying salt tolerance in cotton genotypes. Understanding how different genotypes respond to salt stress and maintain ion homeostasis is crucial for developing salt-tolerant cultivars in the future.

Under salt stress conditions, the growth parameters of seedlings, including the fresh and dry weights of roots and shoots, showed a declining trend. Importantly, the salt-sensitive genotype GP2 exhibited a more significant growth reduction than the salt-tolerant genotype NY2. This observation can be attributed to the detrimental effects of oxidative stress, ion toxicity, disruption of metabolic processes, and osmotic imbalances triggered by salt-induced stress [54,61]. The NY2 genotypes, on the other hand, demonstrated higher values of comprehensive salt tolerance index (CSTI) and relative water content (RWC) compared to the GP2 genotypes. Barley has already shown a decrease in relative water content due to salt stress, which is consistent with the findings of this investigation [62]. Previous research has suggested that these physiological characteristics can be reliable biomarkers for plant salt tolerance screening [63,64]. The improved CSTI and RWC values in the NY2 genotypes indicated their enhanced capacity and efficiency in water uptake, which helps to mitigate tissue desiccation in stress-tolerant plant species [65]. The findings of this study suggest that the regulation of water absorption and distribution is a prevalent and early response in salt-tolerant and salt-sensitive cotton varieties following the detection of salt stress signals originating from the roots. These findings contribute to the broader body of knowledge on plant responses to salt stress and provide a foundation for further research to improve crop resilience in saline environments.

### 3.2. Comparative Analysis of Transcriptome Data and Differentially Expressed Genes Related to Salt Tolerance

Understanding the molecular mechanisms underlying salinity tolerance is critical for developing salt-tolerant cultivars through molecular breeding techniques in cotton. In recent years, transcriptome data and RNA sequencing analysis have provided valuable insights into the molecular and biological processes involved in plant stress responses. RNA-seq is a simple, fast, and cost-effective method that has enabled the identification and characterization of stress-responsive genes in various plant species, including wild barley, rice, eggplant, sweet potato, pearl millet, and soybean [18,23,23,66,67,68,69,70,71,72]. Our results suggested that the salt-tolerant genotype regulates fewer genes to counteract the detrimental effects of salt, while the salt-sensitive genotype activates a larger number of genes in response to survival needs under salt stress conditions. This finding aligns with previous research conducted on rice and eggplant [68,73].

### 3.3. MAPK, SSM, PPI, FB, PHST, and FA Pathways Play Role in Salt Stress Tolerance

Transcriptome analysis revealed several key pathways in the plant’s response to salt stress. Specifically, mitogen-activated protein kinase (MAPK), plant-pathogen interaction (PPI), starch and sucrose metabolism (SSM), plant hormone signaling pathway (PHST), photosynthesis, and fatty acid metabolism (FAM) pathways were found to be transcriptionally reprogrammed in response to salt stress. The significant transcriptional reprogramming observed in these pathways provides evidence of their involvement in the plant’s response to salt stress. Understanding the regulation and function of genes within these pathways can contribute to developing strategies for enhancing salt tolerance in plants [74,75,76]. Studies also exhibited that pathways between PPI, SSM, PHST, and FAM played crucial roles in molecular regulatory mechanisms of salt stress [77,78,79]. Our finding suggests that MAPK cascades play a significant role in the transduction of salt stress signals. The Kyoto Encyclopedia of Genes and Genomes (KEGG) pathway revealed that a significant proportion of the differentially expressed genes (DEGs) in cotton are associated with salt signaling and transduction pathways. These pathways can be further classified into two main functional categories: MAPK signaling and hormone signal transduction. The present findings showed a conclusion consistent with the study conducted by Goyal et al. [80], wherein a transcriptomic analysis in wheat revealed a correlation between hormone signal transduction and salt tolerance. In the case of two cotton varieties, the number of DEGs observed under salt stress conditions was notably greater than that observed under normal conditions. These results align with previous transcriptome investigations conducted on barley under salt stress [81].

### 3.4. TF, RLK, CAZy, and Reported Salt Tolerance-Related Genes

Transcription factors (TFs) play a crucial role in plants’ responses to salt stress by regulating downstream gene expression [82,83]. We identified several TFs belonging to different families, including HD-ZIP (Gh_A11G107200), NAC (Gh_D13G165700), ERF (Gh_D05G245400), R2R3-MYB (Gh_A06G146700), and C2H2 (Gh_A12G020100), that were upregulated to confer salinity tolerance. At the same time, bZIP (Gh_D09G076500) and HSF (Gh_A06G070600) showed upregulation at 3, 12, and 24 h. Previous studies have reported significant changes in the expression of these TFs in response to salt stress, highlighting their involvement in the salt stress response [84,85,86,87,88]. These TFs regulate various mechanisms, such as hormone signaling, activation of salt-tolerant genes, regulation of intracellular ROS levels, and maintaining ion balance to cope with salt stress [89,90]. In diploid wild cotton species, exposure to salt stress had a specific impact on the WRKY, NAC, AP2, bHLH, bZIP, and MYB families of transcription factors within 12 to 24 h after the onset of stress. Additionally, it was observed that most of the identified transcription factors exhibited significant expression levels between 96 and 142 h following the initiation of stress [91]. Upregulation of receptor-like kinase genes was observed by SD-2b (Gh_D10G199300, Gh_D10G199200), L-LEC (Gh_D10G026800), DLSV (Gh_D01G031000), LysM (Gh_D11G049400), and CrRLK1L-1 (Gh_D07G198500). RLCK (Gh_A11G195200) is considered a novel gene because it was upregulated in the NY2 genotype but downregulated in GP2. The findings correlate with prior studies, indicating that a significant quantity of RLKPelle proteins were linked to both biotic and abiotic stresses in Arabidopsis, rice, and barley [92], as well as receptor-like kinases being involved in salinity tolerance by regulating stress-responsive genes [93].

Carbohydrate-active enzymes (CAZy Family), including glycosyltransferases (GTs) and glycoside hydrolases (GHs), also play a role in plants’ responses to salt stress. Expression patterns of carbohydrate-active enzymes highlighted that GH36 (Gh_A05G362500), GT8 (Gh_D13G204000), GH18 (Gh_D13G213600), GT20 (Gh_D08G093700), and CBM18 (Gh_A10G141200) were upregulated in each comparison group while GT1 (Gh_D13G179300) and GT43 (Gh_D06G029900) were upregulated at 3, 12, and 24 h. Our investigation revealed higher expression levels of GT and GH genes in *G. purpurascens*, associated with improved salt tolerance in Arabidopsis [94,95]. These enzymes participate in various catalytic processes, such as sugar conversion and interactions with other molecules, contributing to plant adaptation to salt stress.

A significant implication of the present study is establishing a direct correlation between a comprehensive RNA-seq dataset and qRT-PCR analysis. This correlation facilitated the identification of key genes linked to salt tolerance in cotton and established a relationship between differentially expressed genes (DEGs) and salt stress tolerance. The upregulation of the Gh_A10G166600 gene in salt-tolerant and salt-sensitive genotypes is attributed to late embryogenesis abundant (LEA) proteins and is a possible candidate gene for salt tolerance in cotton. LEA proteins are low-molecular-weight proteins (10–30 kDa) that protect higher plants from environmental stresses [96]. LEA proteins play a role in preventing protein inactivation and aggregation by acting as a molecular shield. This shield impedes the interaction between unfolded proteins, thereby maintaining their functional integrity.

Additionally, LEA proteins may be involved in ion sequestration. Furthermore, it is hypothesized that the adoption of an amphipathic α-helical conformation in the desiccated state aids in the incorporation of certain LEA proteins into the lipid bilayer, thereby serving to inhibit membrane fusion [97]. Similar investigations illustrated that the accumulation of LEA proteins occurs during the growth arrest of rice seedlings induced by salinity, and their mobilization occurs during seedlings’ recovery from salinity-induced stress [98]. Regarding our findings, LEA proteins have also been reported salt-tolerant genes in mulberry [99], jatropha [100], citrus [101], and tobacco [102].

The mechanisms of plant salt stress responses are regulated by intricate signaling and transduction pathways that involve cross-talk between various components [103,104]. Our analysis of differentially expressed genes supports the involvement of the MAPK signaling pathway, plant-pathogen interaction, starch and sucrose metabolism, plant hormone signaling pathway, photosynthesis, and fatty acid metabolism in mediating the response to salt stress [74,75,76]. The results presented herein are consistent with previous transcriptome investigations conducted on barley under salt stress conditions [81]. The transcriptome findings align with empirical physiological data, indicating stronger expression of responsive genes in the salt-tolerant cotton variety than in the salt-sensitive one.

## 4. Materials and Methods

### 4.1. Plant Materials

Forty-five *Gossypium purpurascens* genotypes were collected from Sansha Island in Hainan province (16.83 N, 112 E), Naozhou Island (20.91 N, 110.59 E), and Techeng Island (219 N, 110 E) of Zhanjiang in Guangdong province, China (Supplementary Table S1). As a comparison, one control variety of *Gossypium hirsutum* (Zhong 9807) known for its high salt tolerance [105] was obtained from the gene bank of the Cotton Research Institute, Chinese Academy of Agricultural Sciences (CRI, CAAS) in Anyang (36.1 N, 114.33 E), Henan province, China. Professor Wuwei Ye identified the salt tolerance characteristics of the control variety Zhong 9807, which have been widely exploited in various research studies [106,107,108].

### 4.2. Plant Growth Conditions and Salinity Stress

To initiate the experiment, cotton seeds were subjected to a treatment process. Firstly, the seeds were immersed in a 15% sodium hypochlorite solution for 15 min. Subsequently, they were thoroughly rinsed five times with double-distilled water (ddH2O) to remove residual chemicals. The treated seeds were then grown in cups containing a sand and water mixture with a ratio of 8:1. This growth phase took place over three weeks in a thermostatically controlled greenhouse. The greenhouse maintained a relative humidity of 70–75% and followed a 14 h light and 10 h dark cycle at Zhengzhou Research Base, State Key Laboratory of Cotton Biology, Zhengzhou University, located in Zhengzhou, China.

At the three-true-leaf stage (3TLS), the plants were subjected to two different concentrations of sodium chloride (NaCl): 0% (control) and 0.4% (treatment). To minimize the immediate impact of salt on the plants, the salt was gradually added with the final concentration reaching 0.4% NaCl. Each experimental group consisted of three replicates to ensure reliable results. Based on the salt resistance evaluation of all varieties, we selected one salt-tolerant (ST) variety K386 (NY2) and one salt-sensitive (SS) variety K396 (GP2) for RNA sequencing. Leaf samples were collected at various time points following the onset of salt treatment, including 0 h, 0.5 h, 3 h, 12 h, and 24 h. The collected samples were immediately stored at −80 °C to preserve their RNA integrity for subsequent experiments.

### 4.3. Physiological and Biochemical Traits

A subset of ten seedlings from both the control and treatment groups were randomly selected to assess plant growth and physiological parameters. Samples of roots and shoots were obtained from each of these selected seedlings. The initial step involved the measurement of the fresh weight (FW) of both the roots and shoots of each seedling. Subsequently, the seedlings were placed in individual paper bags, ensuring proper labelling for identification. The bags containing the seedlings were then transferred to an oven set at 105 °C and subjected to drying for 30 min. This initial drying step helped to remove surface moisture from the plant tissues. The seedlings were then dried at a constant temperature of 80 °C until a stable weight was achieved. Following the cooling process, the shoots’ and roots’ dry weights (DW) were determined and soon after, the root-shoot ratio and water content were computed (Formulas (1) and (2)).

(1) $$\mathrm{Relative}\;\mathrm{Water}\;\mathrm{Content}\;\left(\mathrm{RWC}\right)=\frac{\mathrm{FW}-\mathrm{DW}}{\mathrm{FW}}\times 100$$

(2) $$\mathrm{Root}-\mathrm{Shoot}\;\mathrm{Ratio}\;\left(\mathrm{RS}\;\mathrm{Ratio}\right)=\frac{\mathrm{DW}\;\mathrm{of}\;\mathrm{the}\;\mathrm{root}}{\mathrm{DW}\;\mathrm{of}\;\mathrm{shoot}}$$

Later, each dried sample was weighed (about 0.2 g), ground, and made into ashes by heating the samples at 550 °C for 12 h. Following microwave digestion in 70% (*v*/*v*) nitric acid, dried samples were examined for Na, Mg, and K contents using inductively coupled plasma-optical emission spectroscopy (Optima 3000DV; PerkinElmer, Wellesley, MA, USA) [109,110]. The ion-selective transport coefficient from root to stem or stem to leaf is as follows (Formula (3)):

(3) $$\mathrm{Ion}\;\mathrm{Selected}\;\mathrm{Coeffecient}\;\left(\mathrm{ISC}\right)=\frac{S\left(L\right)\left(\frac{K}{\mathrm{Na}}\right)}{R\left(S\right)\left(\frac{K}{\mathrm{Na}}\right)}$$

To evaluate the salt tolerance of the 46 cotton varieties, the comprehensive salt tolerance index (CSTI) of each variety was obtained using associated functions and principal component analysis (PCA) [111]. In short, the ratio of traits evaluated during salt treatment to traits measured under control conditions (salt tolerance index (STI) or relative value) was calculated first. Then we calculated the membership function according to Formula (4). Due to the negative correlation between Na+ and salt tolerance, Formula (5) must be used to calculate the related functions. Then, principal component analysis was used to examine the STI of each variable using the factominer package [112]. Finally, eigenvalues of the 80% contribution rate calculated the load score of each major component by multiplying it by the membership function value to obtain the comprehensive PC score (Table S1A,B).

(4) $$\mu \left(STIi\right)=\frac{STIi-STImin}{STImax-STImin}\;\;\;\;\;\;\;\;\;\;i=1,2,3,\ldots \ldots ,n$$

(5) $$\mu \left(STIi\right)=\frac{STImax-STIi}{STImax-STImin}\;\;\;\;\;\;\;\;\;i=1,2,3,\ldots \ldots ,n$$

where *STIi* designates the *i*th salt tolerance index for a genotype, and *STImin* and *STImax* indicate the minimum and maximum values among *i* salt tolerance indexes, respectively.

(6) $$W_{i}=\frac{P_{i}\;}{\sum_{i=1}^{n}Pi}\;\;\;\;\;\;\;\;\;i=1,2,3,\ldots \ldots ,n$$

where *W_i_* indicates the weight of component *i*’s contribution to the total variance percentage.

Finally, the CSTI values were generated using the following equations:

(7) $$\mathrm{CSTI}=\sum_{j=i}^{n}\left[\mu \left(CSIi\times Wi\right)\right],\;\;\;i=1,2,3,\ldots \ldots ,n\;$$

where *W_i_* indicates the weight of component *i*’s contribution to the total variance percentage.

### 4.4. Transcriptome Profiling

To investigate the plants’ transcriptional response to salt stress, we conducted transcriptome profiling using the Illumina HiSeq platform. Leaf samples were collected from two selected genotypes, with three replicates for each genotype and treatment combination. Total RNA was extracted from the samples using TRIzol reagent, and the quality and integrity of the RNA were assessed using 1% agarose gel electrophoresis and the Agilent 2100 Bioanalyzer system (Agilent Technologies, Palo Alto, CA, USA). We generated paired-end reads for sequencing and performed quality control to remove low-quality and short reads using FastQC (Version 0.11.9) and a Perl program. Gene expression levels were quantified using featureCounts, and the expected number of fragments per kilobase of transcript per million mapped reads (FPKM) was calculated for each gene based on the read counts.

Differential expression analysis was conducted using the DESeq R package (Version 1.34.1), applying a significance threshold of FDR (false discovery rate) ≤ 0.05. Genes were considered differentially expressed if they exhibited at least a 2-fold difference in FPKM values between the compared samples. This analysis allowed us to identify genes that showed significant changes in expression levels in response to salt stress. Transcriptome profiling was employed to comprehensively understand the alterations in gene expression linked to salt stress in the examined genotypes. This approach enabled us to uncover potential candidate genes and pathways involved in the plants’ response to salt stress and to compare the transcriptional profiles between salt-tolerant and salt-sensitive genotypes.

### 4.5. Library Preparation for Transcriptome Sequencing

Following the completion of quality inspection, a quantity of 1.5 μg RNA was extracted from each sample, and three replicates were established for library construction. The NEBNext^®^ Ultra™ RNA Library Prep Kit (NEB, Ipswich, MA, USA) was utilized to create libraries under the manufacturer’s guidelines. The library fragments were purified using the AMPure XP system (Beckman Coulter, Beverly, MA, USA). Subsequently, polymerase chain reaction (PCR) was executed utilizing 3 μL of uracil-specific excision reagent (USER) enzyme procured from New England Biolabs (NEB), United States of America (USA), in conjunction with the complementary DNA (cDNA) at a temperature of 37 °C for 15 min, followed by 95 °C for 5 min. Subsequently, the PCR products underwent purification via the AMPure XP system, and the quality of the library was assessed utilizing the Agilent Bioanalyzer 2100 system. The sequencing of libraries was conducted using an Illumina Novaseq 6000 platform provided by Beijing Allwegene Technology Company Limited in China. The sequencing adapters were removed from the raw data and low-quality reads were eliminated to obtain clean reads. The reference genome employed in this study was *Gossypium hirsutum*, as reported by [113]. The reference genome’s index was generated through the utilization of Bowtie v2.2.3. Subsequently, the paired-end cleaned reads were aligned to the reference genome through TopHat v2.0.12. The fragments per kilobase of exon model per million mapped reads (FPKM) were computed for each gene using HTSeq v0.6.1 to map the genes, and the gene-specific read counts were assigned. Subsequently, the expression level of the gene was evaluated.

### 4.6. Differential Expression Analysis of Salt-Tolerant Genes

DESeq2 was utilized to conduct differential expression analysis (DEGs) between two treatments [114]. The criteria for determining significant differential expression were FDR < 0.05 and a fold change of at least 2. The GOseq R packages were utilized to conduct a gene ontology (GO) enrichment analysis on the differentially expressed genes (DEGs) set. KEGG significant enrichment analysis was performed (Q-value < 0.05) https://www.omicshare.com/tools/ (accessed on 1 April 2023). To further analyze the specific genes related to salt tolerance between the two genotypes, we established the following strict screening parameters: (1) at four salt treatment time intervals, only genes will be selected with differential expression found between NY2 and GP2 (i.e., NY2 vs. GP2-05/3/12/24 DEGs); (2) only differentially expressed genes in salt-tolerant varieties will be selected (i.e., four comparative groups’ DEGs of NY2-05/3/12/24 vs. 0); (3) in both salt-tolerant and salt-sensitive varieties, there are differentially upregulated genes, but the difference in log2 fold change will be greater than 0.6 (i.e., log2NY2-05/3/12/24 vs. 00-log2GP2-05/3/12/24 vs. 00 > 0.6); (4) in both salt-tolerant and salt-sensitive varieties, there are differentially downregulated genes, but the difference in log2 fold change will be less than −0.6 (i.e., log2GP2-05/3/12/24 vs. 00-log2NY2-05/3/12/24 vs. 00 < −0.6). The specific screening process, gene count statistics, and final results are all included in the Supplementary Figure S3 and Table S3.

### 4.7. Construction of Weighted Gene Co-Expression Network (WGCNA)

R programming was used to analyze weighted gene co-expression networks with the help of the WGCNA package [115]. FPKM > 1 and a normalized read counts matrix were used to evaluate 29 transcriptome samples. For WGCNA, all differentially expressed genes with major variations were selected. The initial scaled relationship matrix underwent power processing to produce an unscaled adjacency matrix after threshold screening with β = 12. Gene clustering and module division were accomplished using the dynamic shearing approach. A module was required to include at least 30 genes (minModuleSize = 30), and 0.25 was the cutoff value for merging similar modules. After performing principal component analysis (PCA) on each module’s genes, the value of principal component 1 (PC1) was referred to as the module eigengene (ME). For screen-specific modules against salt resistance, the correlation coefficient r and accompanying *p*-value were determined between the module eigenvectors of each module and various samples. Positive and negative correlations were shown by correlation coefficients with r > 0 and r < 0, respectively. The specificity modules in this study were further examined as |r| > 0.6 and *p* modules < 0.05. The genes corresponding to the top five weight values of their expression correlation were retrieved after the target genes with the strongest correlation coefficient among modules were sorted out. The WGCNA graph was constructed using the Gephi-0.91 program [116]. Utilizing a gene regulatory relationship network map facilitated the accurate identification of potential candidate genes that may engage in regulatory interactions with the target genes. This map can also be employed to predict the functions of unknown genes by leveraging the functions of known genes.

### 4.8. Quantitative Real-Time PCR Analysis

Quantitative real-time PCR (qRT-PCR) analysis was conducted to measure the expression levels of 18 genes associated with salt tolerance in NY2 and GP2 at 0.5, 3, 12, and 24 h. The first set of qRT-PCR cDNA was produced in a 20-mL solution, which included 1 mg of RNA as the template. The First-strand cDNA Synthesis SuperMix kit (No. E047-01B; Novoprotein, Shanghai, China) was utilized for this purpose, and the manufacturer’s instructions were followed. Subsequently, the Novostar R SYBR qPCR SuperMix Plus (Code No. E096-01A; Novoprotein, Shanghai, China) was utilized to conduct qRT-PCR on a LightCycler 480 (Roche, Mannheim, Germany). The gene used as a housekeeping gene in this study was identified as GhHistone. The Primer-BLAST online tool2 was utilized to design gene-specific primers (Supplementary Table S6). The qRT-PCR analysis was conducted in triplicate for each biological replicate, with each technical replicate also performed in triplicate. The gene expression values were determined using the 2^−ΔΔCt^ approach [117]. Here, we calculated the relative expression levels of control sample (CK) genes first, and one of the biological replicates was identified as 1 during calculation, while the other two biological replicates were greater than or less than 1. According to the 2^−ΔΔCt^ method, the relative values of the stress samples at different time points can be obtained.

### 4.9. Data Analysis

Microsoft Excel 2021 was utilized to statistically analyze various phenotypic markers and determine their salt tolerance value. IBM SPSS 25.0 was employed to conduct morphological, physiological, and biochemical data analyses (IBM Corp., Armonk, NY, USA). Principal component analysis and cluster analysis were performed using R programming. Two-way ANOVA determined the significance of the phenotypic trait data between the two cotton genotypes, and qRT-PCR results were presented with GraphPad Prism 9.0 (GraphPad Software, Inc., San Diego, CA, USA). Each experiment was repeated at least three times, and the values were expressed as mean ± SE.

## 5. Conclusions

In conclusion, this study utilized RNA-seq analysis to explore the molecular mechanisms underlying salt tolerance in two *G. purpurascens* cultivars at the seedling stage. This study identified one salt-tolerant variety and one salt-sensitive variety based on 34 traits and CSTI values in wild cotton species. Transcriptome analysis revealed 2776, 6680, 4660, and 4174 salt-tolerant differentially expressed genes at 0.5, 3, 12, and 24 h, respectively, and pathways associated with salt resistance by comparing the transcriptome data of salt-tolerant and salt-sensitive genotypes. GO and KEGG pathway enrichment analysis revealed the involvement of various biological processes, including the MAPK signaling pathway, plant-pathogen interaction, starch and sucrose metabolism, the plant hormone signaling pathway, photosynthesis, and fatty acid metabolism, in response to salt stress. ERF, bHLH, and WRKY were key transcription factors in salt tolerance. WGCNA revealed that metabolic pathways and MYB4, MYB36, MYB43, MYB105, bZIP16, and bZIP43 played significant roles in salinity tolerance. Furthermore, qRT-PCR highlighted the sensitivity of Gh_A10G166600 to salt stress and its potential synergistic relationship with LEA proteins in mitigating the harmful effects of salinity. The findings contribute to our understanding of the genetic basis of salt tolerance in cotton and provide valuable genetic resources for future salt-resistant breeding efforts. This pioneering research expands our knowledge of salt stress responses in *Gossypium purpurascens* and lays the foundation for developing improved salt-tolerant cotton varieties.

## Data Availability

The datasets presented in this study can be found in online repositories. The transcriptome sequencing data have also been deposited in the National Genomics Data Center under accession number CRA011383 (https://bigd.big.ac.cn/gsa/browse/CRA011383; accessed on 20 June 2023).

## Supplementary Materials

The following are available online at https://www.mdpi.com/article/10.3390/ijms241612853/s1.
