# Supplementary material for: Comprehensive Evaluation and Transcriptome Analysis Reveal the Salt Tolerance Mechanism in Semi-Wild Cotton (Gossypium purpurascens)
Source: Int J Mol Sci. 2023 Aug 16;24(16):12853. doi: 10.3390/ijms241612853 (PMC10454576; doi:10.3390/ijms241612853)
Supplement: Supplementary file 1 [file ijms-24-12853-s001.zip › Figure S1.pdf]

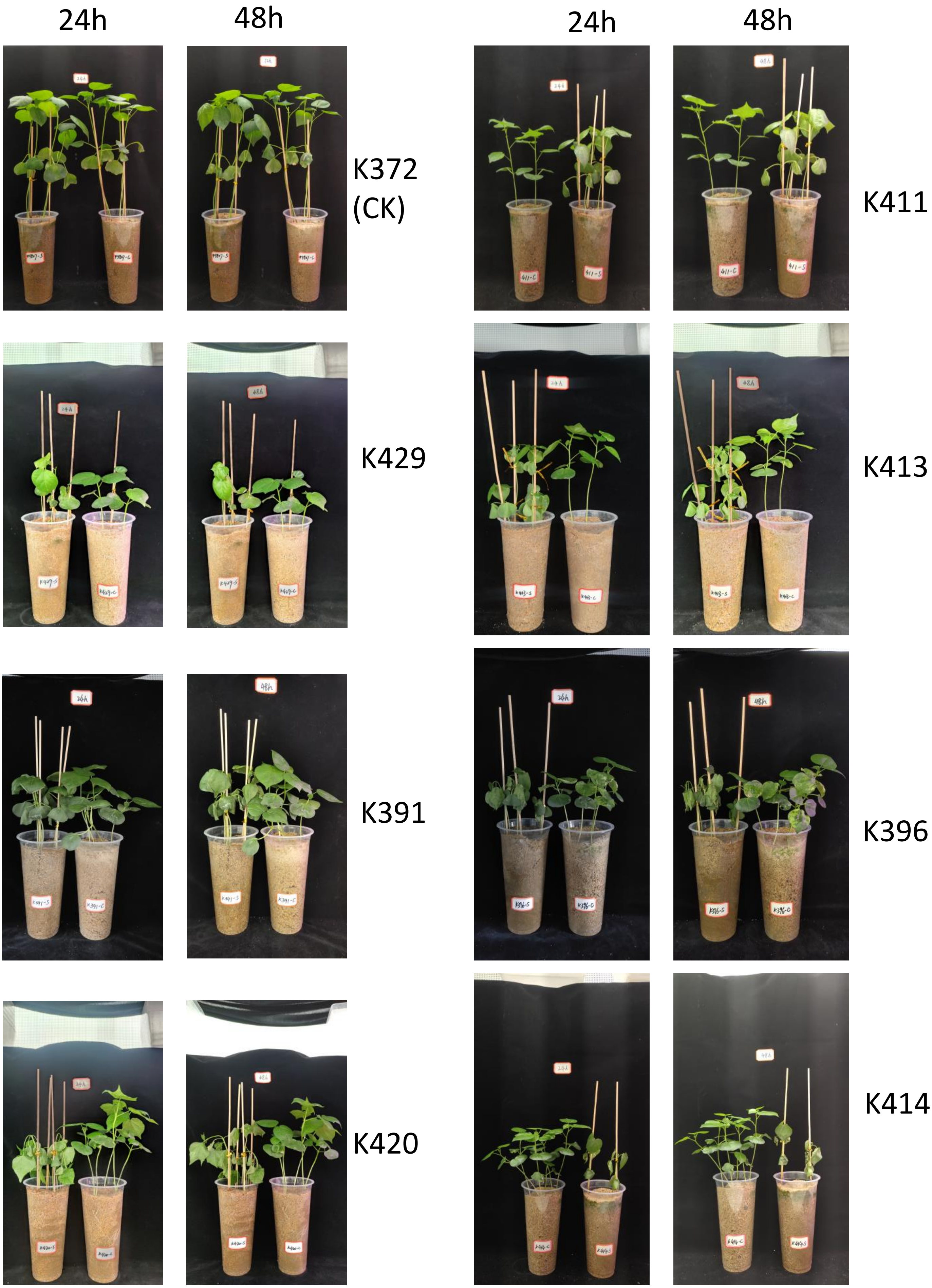

Figure S1:Phenotypic validation of salt tolerance evaluation of *Gossypium purpurascens.* Note*:* K372(Zhong9807) is upland cotton as CK.
