# Supplementary material for: Comprehensive Evaluation and Transcriptome Analysis Reveal the Salt Tolerance Mechanism in Semi-Wild Cotton (Gossypium purpurascens)
Source: Int J Mol Sci. 2023 Aug 16;24(16):12853. doi: 10.3390/ijms241612853 (PMC10454576; doi:10.3390/ijms241612853)
Supplement: Supplementary file 1 [file ijms-24-12853-s001.zip › Figure S2.pdf]

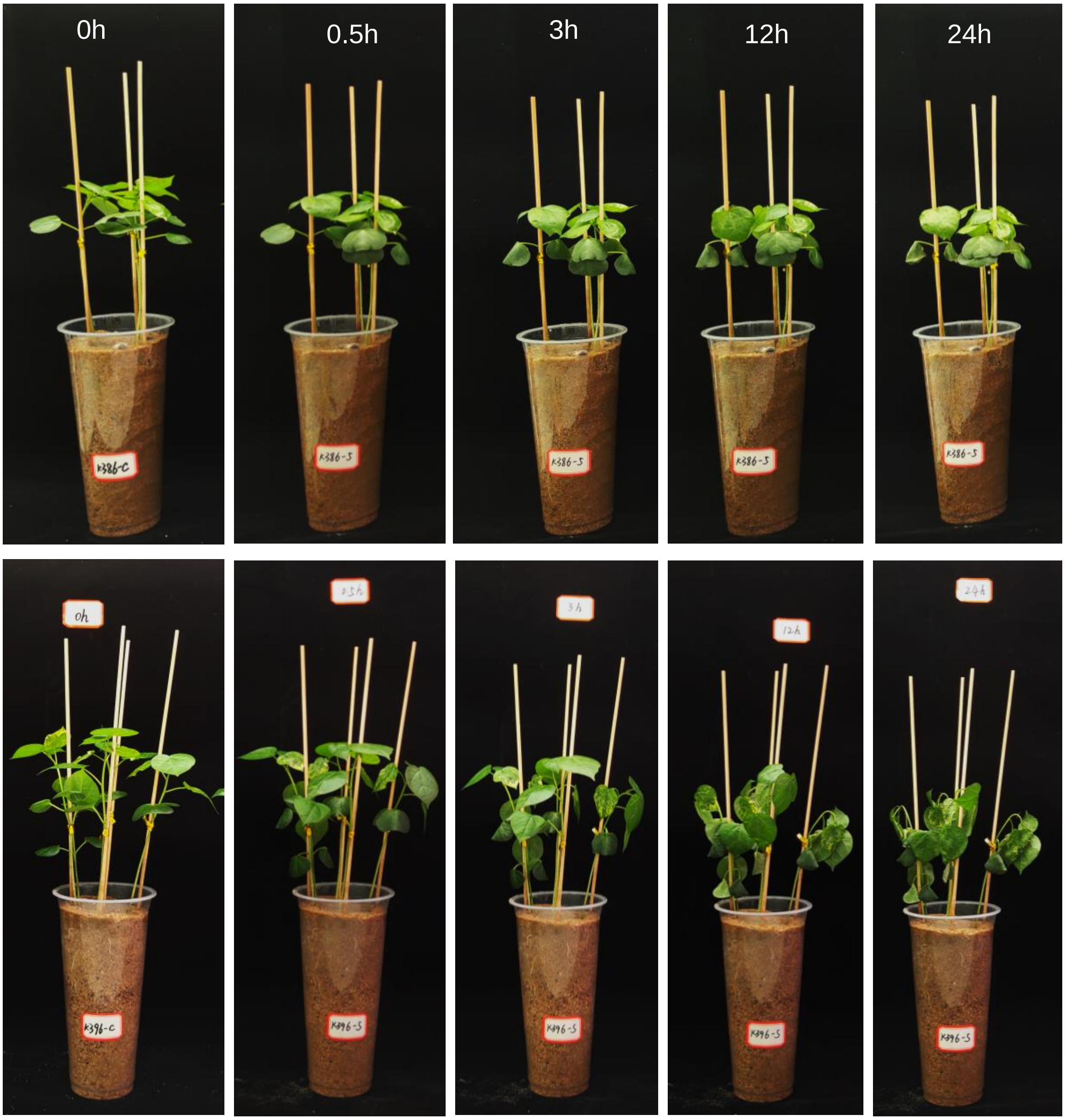

Figure S2:Response performance of two salt tolerant and sensitive germplasm under salt stress at 0.5.3.12.24 hours
