# Supplementary material for: Comprehensive Evaluation and Transcriptome Analysis Reveal the Salt Tolerance Mechanism in Semi-Wild Cotton (Gossypium purpurascens)
Source: Int J Mol Sci. 2023 Aug 16;24(16):12853. doi: 10.3390/ijms241612853 (PMC10454576; doi:10.3390/ijms241612853)
Supplement: Supplementary file 1 [file ijms-24-12853-s001.zip › Figure S4.pdf]

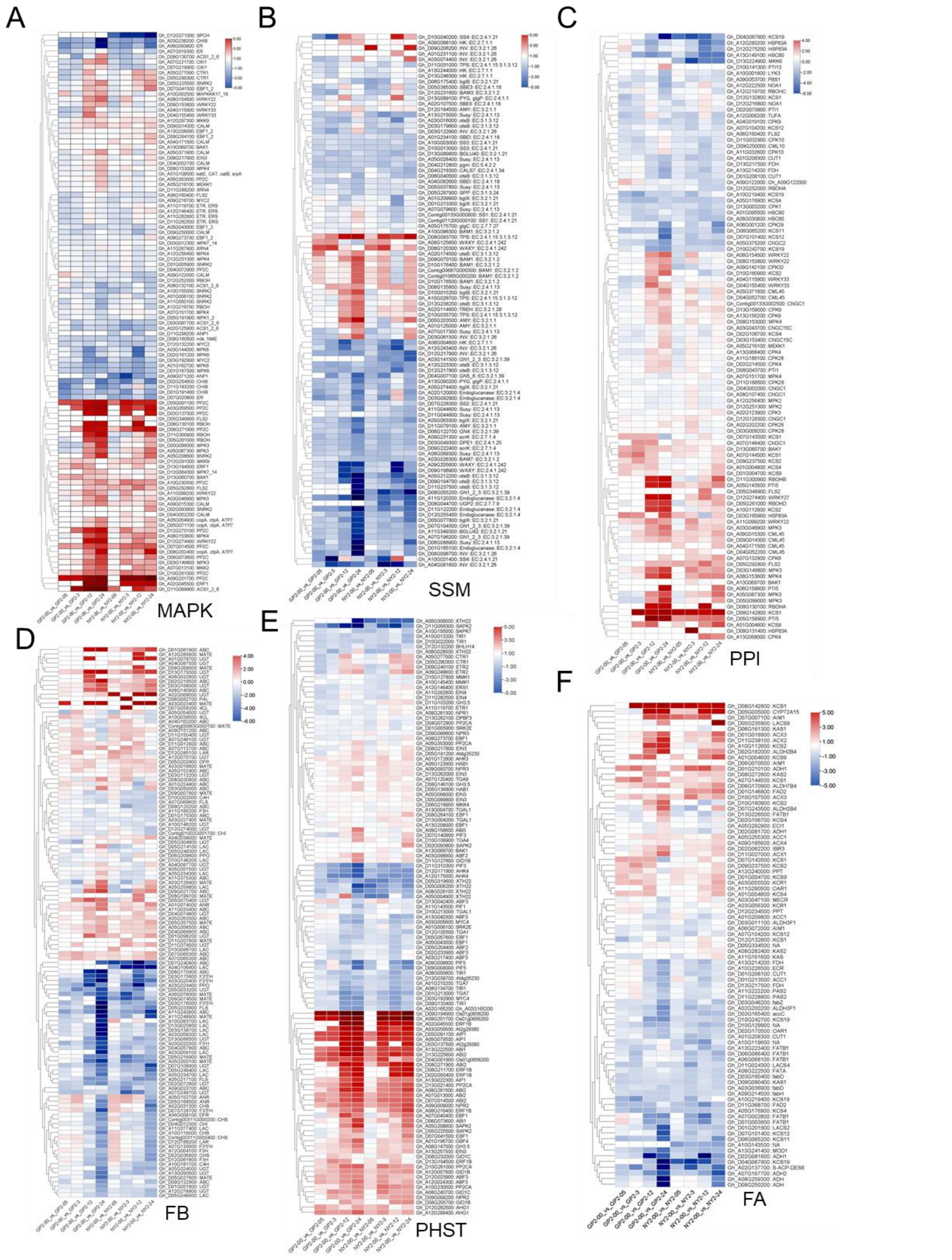

Figure S4: Heat map of differential expression of upregulated and downregulated genes in the KEGG metabolic pathway related to salt tolerance.(A) MAPK signaling pathway;(B) Starch and sucrose metabolism;(C) Plant-pathogen interaction;(D) Flavonoid biosynthesis;(E) Plant hormone signal transduction;(F) Fatty acid metabolism &elongation
