# Supplementary material for: Comprehensive Evaluation and Transcriptome Analysis Reveal the Salt Tolerance Mechanism in Semi-Wild Cotton (Gossypium purpurascens)
Source: Int J Mol Sci. 2023 Aug 16;24(16):12853. doi: 10.3390/ijms241612853 (PMC10454576; doi:10.3390/ijms241612853)
Supplement: Supplementary file 1 [file ijms-24-12853-s001.zip › Figure S5.pdf]

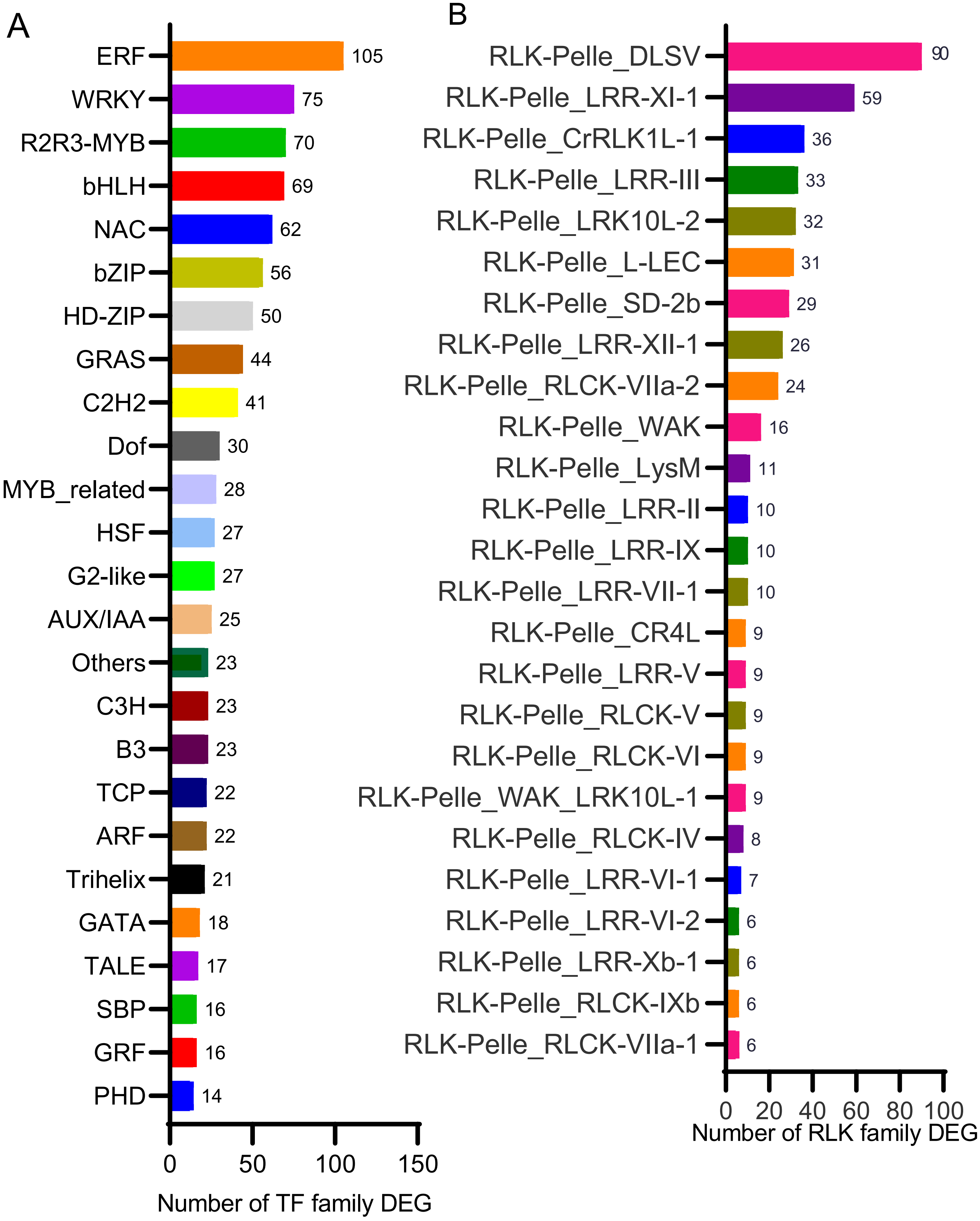

Figure S5: Differential expression analysis of genes related to salt tolerance under salt stress.(A) transcript factors (TFs) family ; (B) Receptor-like kinases (RLKs) family.
