# Supplementary figures and images for: Comprehensive Evaluation and Transcriptome Analysis Reveal the Salt Tolerance Mechanism in Semi-Wild Cotton (Gossypium purpurascens)
Source: Int J Mol Sci. 2023 Aug 16;24(16):12853. doi: 10.3390/ijms241612853 (PMC10454576; doi:10.3390/ijms241612853)

05h

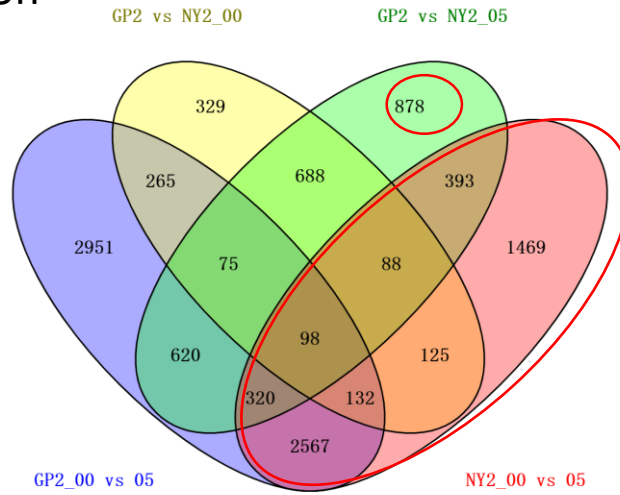

3h

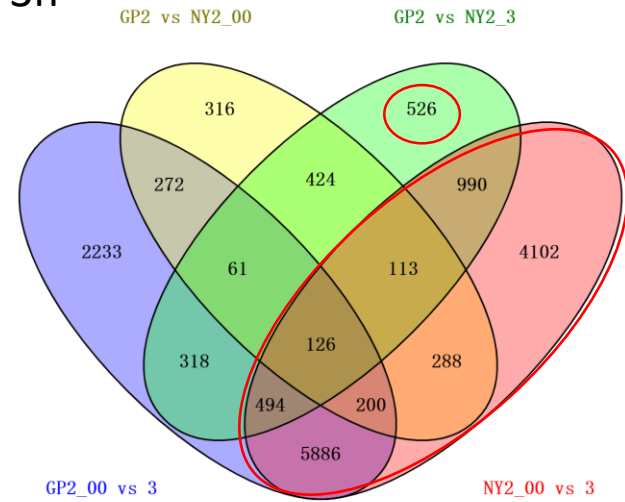

12h

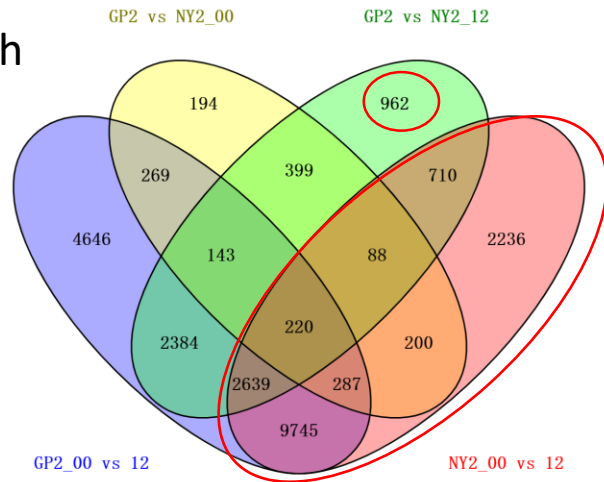

24h

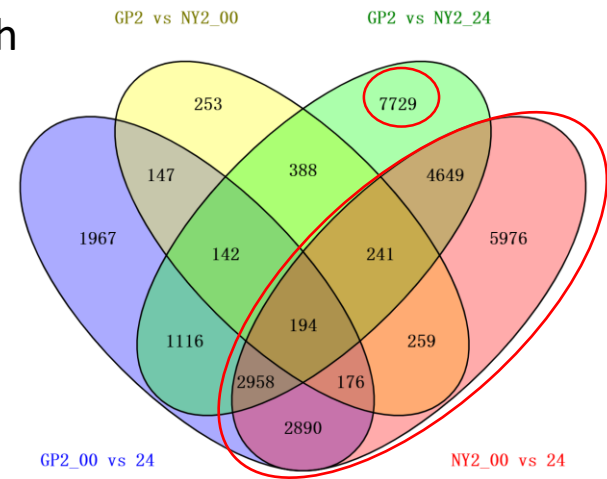

Fig S3: Screening strategy for salt tolerant genes (only selecting NY2 vs GP2 DEG & NY2 CK vs T)

Supplement: Supplementary file 1 [file ijms-24-12853-s001.zip › Figure S3.pdf]
